# Imagining Sisyphus happy: DNA barcoding and the unnamed majority

**DOI:** 10.1098/rstb.2015.0329

**Published:** 2016-09-05

**Authors:** Mark Blaxter

**Affiliations:** Institute of Evolutionary Biology, University of Edinburgh, Edinburgh EH9 3FL, UK

**Keywords:** metabarcoding, Sisyphus, meiofauna

## Abstract

The vast majority of life on the Earth is physically small, and is classifiable as micro- or meiobiota. These organisms are numerically dominant and it is likely that they are also abundantly speciose. By contrast, the vast majority of taxonomic effort has been expended on ‘charismatic megabionts’: larger organisms where a wealth of morphology has facilitated Linnaean species definition. The hugely successful Linnaean project is unlikely to be extensible to the totality of approximately 10 million species in a reasonable time frame and thus alternative toolkits and methodologies need to be developed. One such toolkit is DNA barcoding, particularly in its metabarcoding or metagenetics mode, where organisms are identified purely by the presence of a diagnostic DNA sequence in samples that are not processed for morphological identification. Building on secure Linnaean foundations, classification of unknown (and unseen) organisms to molecular operational taxonomic units (MOTUs) and deployment of these MOTUs in biodiversity science promises a rewarding resolution to the Sisyphean task of naming all the world's species.

This article is part of the themed issue ‘From DNA barcodes to biomes’.

I hope there's an animal somewhere that nobody has ever seen. And I hope nobody ever sees it.

Wendell Berry ‘To the unseeable animal’ [1].

## 1. The Linnaean revolution: biology's enduring megaproject

Physics and astronomy are replete with megaprojects, such as the Large Hadron Collider and the Mars missions, that dwarf other modern investments in basic science. But these projects, and the recent huge projects in biology—ENCODE [2], the Human Genome Project [3], the Structural Genomics project [4]—are dwarfed by the longest-running, most successful and most impactful biology megaproject of all: the 263-year old Linnaean project [5,6]. The revolution in biology initiated by Linnaeus in proposing a binomial system for ‘naming’ groups of plants and animals that were recognizable as distinct natural types has changed the world. These names are a *lingua franca* that can be used to communicate complex concepts and understanding across the globe, helping to organize agriculture, trade and industry, and that is the standard against which we can measure human impact on the planet's ecosystems. The project has been delivered by hundreds of thousands of taxonomists, working lifetimes to describe an estimated 1.2 million species [7]. For every krona, euro, dollar or yen invested in taxonomy, the world economy has likely reaped many-fold returns through identification of disease organisms, invasive species, important biomaterial sources and biomarker taxa.

But how many species of life are there on the Earth? And how close are we to completing the great catalogue? Through careful modelling of species diversity in different groups and encompassing both terrestrial and marine ecosystems, Mora *et al.* [8] estimated that there are 8.7 million species on the Earth, or seven times as many as have been described in the first quarter century of the Linnaean Project. Their estimate, based on careful modelling of known and predicted species numbers in different phyla and kingdoms, sits near the middle of previous estimates [9,10]. Most of the species (7.7 million) are predicted to be Metazoa, and most of the Metazoa are Arthropoda. The catalogue is not complete. As Mora *et al.* [8] said: ‘In spite of 250 years of taxonomic classification and over 1.2 million species already catalogued in a central database, our results suggest that some 86% of existing species on Earth and 91% of species in the ocean still await description.’

The nature of Linnaean taxonomy is such that naming a new species takes a finite amount of effort, but this effort increases as the Linnaean catalogue becomes more complete. Each proposal for new species must be carefully and precisely placed with reference to existing knowledge. Specific diagnosis has to be offered, and the literature and specimens that underpin the diagnoses of closely related taxa must be minutely examined. Naming the remaining 86% of species is going to become ever more difficult, and even with accelerated publication and digitally available descriptions is likely to require a millennium or more of focused effort. It is thus unlikely that the Linnaean catalogue will ever be complete. This is not to say that it is credible that the Linnaean project should stop, but rather that we cannot expect this Sisyphean task to be completed.

For his continued lack of respect for the order of the gods, Sisyphus was condemned for eternity to roll an immense boulder up a steep hill. Each time he neared the summit, and was about to release the boulder to roll down the other side to freedom, the boulder escaped his grasp and thundered back to the plain. Sisyphus had to return to the foot of the hill to restart his task, forever [Homer Odyssey 11.13]. For Linnaean species description, the near-endless task might be cut short by mass extinction.

## 2. DNA sequencing, barcoding and species description

Molecular data have been included in the primary descriptions of new taxa for 20 years or more. For example, in 1996, the first description [11] of the nematode *Pristionchus pacificus*, a species now used as a genetic, developmental and ecological model organism [12], included nuclear small subunit ribosomal RNA sequence. These sequences that accompany the primary species descriptions have been christened ‘genetypes’ [13]. In bacterial taxonomy, molecular data are necessary for species definition, and soon species descriptions for eukaryotic taxa will also include whole genome data. In a retrofit operation, the taxonomy curators at the National Center for Biotechnology Information's GenBank database have been assigning the label ‘sequence type’ to sequence accessions where there is clear evidence that they derive from a submitted type culture or specimen [14]. This NCBI effort now includes (April 2016) over 25 000 taxa, with 13 219 Bacteria, 509 Archaea and 11 471 Eukaryota (of which 1141 are Metazoa). The Metazoan species are largely associated with cytochrome oxidase 1 (COI) barcode submissions. A global effort to determine genome sequences for bacterial type strains is underway [15,16]. These data are significantly augmented by the DNA barcode data amassed by the DNA barcoding community and presented in the BOLD System database [17]. BOLD DNA barcode data are associated (in the main) with specimens, the majority of which in turn have expert morphological species identifications. Thus, sequence-species links are available for over 250 000 Linnaean species of Viridiplantae, Metazoa and Fungi (and approximately 1000 species from four phyla of protists).

## 3. Neglected animals are largely small

Numerically, the overwhelming majority of individual organisms are microscopic, with major body axes less than 1 mm. While this size class obviously includes (most) Bacteria, Archaea and protists, it also includes the majority of Metazoa, a group famed for its charismatic megataxa. For example, in beach sediments the meiofauna outnumber the indwelling meso- and megafauna by orders of magnitude [18]. However, these massive populations might have a small impact on overall diversity if they include relatively few distinct species. Species number estimates for phyla that are largely meiofaunal, for example, those of Mora *et al.* [8], suggest relatively low total species counts, based on low counts of described species. These estimates can be at odds with focused analyses carried out on smaller branches of the tree of life.

Underestimation of species numbers is particularly prevalent in meiofauna, where lack of easily recognized morphological characters (when the whole specimen is 200 µm long, species specific characters may be at the limit of light microscopy), the incompleteness of early descriptions, the variance and environmental plasticity of form, and the sheer abundance of individuals challenge the working methodologies of systematists. In phylum Nematoda, there are approximately 24 000 described species. Hallan, in a painstaking encyclopaedic effort completed in 2007, catalogued 22 136 species level taxa [19], and nematode taxonomists have been busy since then adding to the catalogue (e.g. 15 new species of *Caenorhabditis* in one paper [20]). However, for Nematoda, the modelling of Mora *et al.* [8] estimated a total species number less than the current described species number. Other authors, using data collected from geographically or ecologically restricted sampling, suggest that true Nematoda species numbers may be anywhere between 1 and 100 million [21–25]. While the upper estimates are deprecated, in particular, owing to a more restricted diversity than expected in deep sea benthos communities [25–27], support for a total of approximately 1 million nematode species is credible. Evidence is also emerging of many cryptic species in Nematoda. For example, the widespread and long-studied *Pellioditis marina* has been shown to be a complex of dozens of reproductively isolated metapopulations (i.e. species) that can be distinguished by DNA sequence and mating tests, but not morphology [28]. A similar finding of extensive crypsis in large nematodes (greater than 1 cm) [29] suggests that crypsis may be a common reason for underestimation of species numbers.

There are likely to be many undescribed meiofaunal species, particularly in marine sediments. As marine enoplid nematode specialist Ashleigh Smythe said ‘Marine nematologists get excited when we find a known species’ (personal communication to M.B. 2016). The high estimates of global nematode species numbers [23] are based not on identification of specimens to Linnaean taxa but of sorting of samples into operational taxonomic units (OTUs), and these OTUs are, in turn, likely to conflate cryptic taxa. Identification of nematodes to species is time consuming and requires a high level of expertise, such that comprehensive nematode species inventories are practically impossible using traditional methodologies [30]. The same set of issues (hyperabundance, hyperdiversity, crypsis and lack of distinguishing morphology) are likely to challenge species identification and diversity assessment in other meiobiotal eukaryotes, such as Tardigrada [31], Oribatida [32] and the many single-celled eukaryote phyla.

## 4. Metabarcoding accesses meiofauna

It is trivial to set a plankton net or sieve a few tens of grams of sediment and collect hundreds of thousands of specimens of meiofauna. Nematodes achieve astounding numerical abundances, up to a record of 27 × 10^6^ m^−2^ in an estuary sediment [33]. Like other animals, meiofaunal species abundances follow overdispersion curves, with a few species present in large numbers and most species present rarely. Sifting by eye through a cloud of swimming larvae or thrashing nematodes for rare novelty is a thankless task, and current methodologies subsample from a preserved sample to estimate taxon abundances rather than to enumerate novelty. By repeated sampling, the likely pattern of taxon abundance and diversity can be estimated, but this is costly in researcher time. Advances in sequencing technologies now permit bulk sampling of barcoding genes from a population without the need to individually separate, amplify and sequence each specimen. This approach, pioneered for analysis of the expected 99% of unculturable prokaryotes and microbial eukaryotes [34–36], has been termed metagenetics, environmental barcoding or metabarcoding [37–39]. While the individual specimens are never seen or assessed for Linnaean taxonomy, it is possible to use their sequences as proxies for both their presence (and perhaps abundance) and systematic affinities. The use of PCR in complex mixtures and the inherent errors and biases of the new sequencing technologies mean that careful filtering for errors and artefacts is essential before robust molecular operational taxonomic unit (MOTU) estimation can be carried out [40,41]. With current technologies that involve amplification before sequencing, formation of chimaeric fragments remains a significant issue [41]. The power of new sequencing technologies (generating tens to hundreds of millions of sequences at a time [37]), advanced computational toolkits [42] and ever faster processors now mean that this task is relatively trivial, and as analysis is algorithmic it can be programmed to run automatically [43].

Using this metabarcoding approach a number of groups have started to probe the meiofauna of sediments and soils to ask what is there and to assess whether the ‘extreme’ assertions of meiofaunal species number are likely to be true. Tens of thousands to millions of sequences from a variety of meiofaunal assemblages have now been determined and analysed as MOTU sets [43–46]. These analyses have many notable features. As expected, Nematoda and Arthropoda dominate marine sediments in terms of numbers of reads and of MOTUs. In the same sediments high numbers of Platyhelminthes taxa [44,46] have been found, against expectation. Flatworms are not often observed in abundance in sediments. The disparity between the morphological and barcoding approaches may be because meiofaunal flatworms must be sampled live for morphological identification, as when preserved they are effectively indistinguishable from detritus. However, DNA extraction is agnostic as to appearance and thus delivers flatworm DNA for amplification and sequencing. Importantly, the DNA barcoding approach was able to affirm the presence of 1000–2500 eukaryote MOTUs per survey [43–46], and classify these robustly by comparison with existing databases of marker genes. The paucity of species-tagged reference sequence data for many phyla sampled in these experiments precludes (in most cases) assignment to species-equivalent in the Linnaean system, but the MOTUs can be robustly assigned to genera or families. This is, in most cases, enough to infer likely life-history characteristics such as mode of feeding.

## 5. Molecular operational taxonomic units as species

DNA barcode data can be used to define OTU. We originally called these MOTU and suggested that MOTUs were useful proxies for ‘species’ level taxa, and could be used in a similar way to OTUs [47,48]. Importantly, the algorithm used for MOTU definition can be explicit, and largely deterministic. This permits both hypothesis testing and interoperability of MOTU analyses. It is possible to aggregate data across studies and robustly synonymize taxa in different datasets, desynonymize cryptic taxa, co-cluster larval and adult specimens, and identify prey-derived sequences from gut or faecal samples. The BOLD System database [17] now includes a clustering of barcode sequences into MOTUs, called BINs (Barcode Identification Numbers), wherein DNA barcode sequences have been clustered [49] to define groups that have congruent internal divergence and are distinct from groups generated from related sequences. The BIN system has been critical in advancing discussion and discovery of new taxa, and provides a working example of a post-Linnaean taxonomic system. Importantly, as BOLD System and the Barcode of Life project generally are specimen-based, BINs can be linked to specimens, and the Linnaean diagnoses of those specimens. Similarly, the UNITE project aims to deliver a MOTU-based clustering of fungal isolate internal transcribed spacer (ITS) sequence linked, where possible, to named taxa [50]. UNITE currently presents 53 891 ‘fungal species hypotheses’ based on ITS data.

But are MOTUs ‘species’? In the early years of the DNA barcoding field, there was animated debate as to whether COI data, and particularly the partial COI target chosen for DNA barcoding, was sufficient to distinguish animal species, and whether it was an honest marker of species membership. This debate has not been resolved, but it is clear that for most animal taxa the COI barcode is good at distinguishing species, and that exceptions to this general success are both real and interesting, but not fatal to the programme as a whole. In animals, for example, COI generally accumulates substitutions rapidly enough so as to be informative between all but the most recently diverged taxa, and population genetic processes serve to assure that the coalescence of the mitochondrial haplotype is usually before coalescence of the nuclear haplotypes in a species. Issues arise where mitochondria have introgressed between species, sometimes because of other cytoplasmic genetic elements such as intracellular symbionts like *Wolbachia* [51]. In complex species groups, joint analyses of nuclear and mitochondrial markers may be necessary to distinguish species [52]. Alternative markers, such as the nuclear ribosomal RNA subunits, also have promise, particularly as it is possible to derive near-universal primer sets that amplify all taxa. However, the greater conservation of ribosomal RNA genes that permits universal primer design also limits the specific diagnosis possible with ribosomal RNA sequence. Closely related taxa can be identical in small ribosomal RNA sequence, or so close as to be indistinguishable from sequencing error [48].

Despite these caveats, specimens assigned to MOTUs or BINs can be used in ecological and other surveys just as would specimens assigned to Linnaean taxa [37,43–46]. Individual MOTUs can be assigned abundances and the presence–absence statistics, samples can be compared both within and between studies, and ecological parameters inferred from interaction with abiotic factors and from inferred taxonomic affinities. For meiofaunal surveys, where morphological taxonomy is unable, for operational reasons, to identify OTU to Linnaean species, it can be impossible to cross-compare different ecosystems because it is not possible to synonomize between ‘*Daptonema* sp. 1’ in one publication and the several unallocated *Daptonema* species identified in a second [53].

## 6. Outlook

Some dung beetles famously collect and roll relatively huge pellets of dung in order to provision their offspring, and are a living model for Sisyphus's task. Like all biological mechanisms, this activity has no greater purpose than an attempt by dung beetle genes to produce additional copies of themselves in the future. Camus was fascinated by the myth of Sisyphus [54], in particular, in the way it perhaps echoed the pointlessness of human existence: no matter how hard Sisyphus worked, no matter how often he nearly reached the summit, he always watched his boulder rolling out of control back down the hillside to the plains. But Camus was interested in Sisyphus as he walked back down to restart his task: what was Sisyphus thinking, what did he feel? During the period of this endless return, Camus suggests we must consider Sisyphus happy: happy that the task is still there to be done [54]. Similarly, while we may never complete the cataloguing and systematizing of life on our planet, it is deeply illuminating to attempt to do so. Knowing the limits of diversity, the edges of the puzzle, may be as important as filling in every piece. Especially, as we experience the sixth great extinction it may be a hollow victory to develop a species or MOTU list for a disappearing ecosystem. That said, metagenetics is, I believe, the only way we are going to glimpse the majority of life on the Earth, and, while we may never see the organism we sequence, we will be able to infer its place in phylogeny, impute its biology and consider its roles in driving ecosystem function.

‘La lutte elle-même vers les sommets suffit à remplir un coeur d'homme. Il faut imaginer Sisyphe heureux.’

[The struggle to the summit in itself is enough to fill the heart. We must imagine Sisyphus happy.]

Albert Camus, *Le Mythe de Sisyphe* [54].
